# Clinicopathological Features and Prognostic Evaluation of UBR5 in Liver Cancer Patients

**DOI:** 10.3389/pore.2022.1610396

**Published:** 2022-11-01

**Authors:** Qi Huo, Junjie Hu, Binfen Hou, Mei Zhao, Xue Han, Yulin Du, Yao Li

**Affiliations:** ^1^ Department of Medical Oncology, The Second Affiliated Hospital of Bengbu Medical College, Bengbu, China; ^2^ Anhui Provincial Key Laboratory of Immunology in Chronic Diseases, Anhui Provincial Key Laboratory of Infection and Immunology, Department of Laboratory Medicine, Bengbu Medical College, Bengbu, China

**Keywords:** prognosis, TCGA, liver cancer, UBR5, YWHAZ

## Abstract

**Background:** Typically, liver cancer patients are diagnosed at an advanced stage and have a poor prognosis. N-recognin 5 (UBR5), a component of the ubiquitin protein ligase E3, is involved in the genesis and progression of several types of cancer. As of yet, it is unknown what the exact biological function of UBR5 is in liver cancer.

**Methods:** A Kaplan-Meier survival curve (OS) was used to examine the effect of UBR5 expression on overall survival based on the TCGA database. To determine the molecular functions of UBR5 in liver cancer, we used the Gene Ontology (GO) and Kyoto Encyclopedia of Genes and Genomes (KEGG) databases. A protein-protein interaction (PPI) network was established for the screening of UBR5-related proteins in liver cancer. Western blot analysis was used to determine the expression levels of UBR5 and YWHAZ (tyrosine 3-monooxygenase/tryptophan 5-monooxygenase activation protein zeta), and in order to detect cell proliferation, an MTT assay was used.

**Results:** The expression of UBR5 in liver cancer patient samples is significantly higher than in adjacent normal tissues. A high level of UBR5 expression was associated with older patients, a higher tumor grade, lymph node metastasis, and poor survival. We discovered YWHAZ with high connectivity, and UBR5 expression correlated positively with YWHAZ expression (*r* = 0.83, *p* < 0.05). Furthermore, we found that elevated UBR5 levels directly correlated with YWHAZ overexpression, and that UBR5 promoted cell proliferation by affecting YWHAZ expression. Additionally, the TCGA databases confirmed that patients with liver cancer who expressed higher levels of YWHAZ had poorer outcomes.

**Conclusion:** This suggests that UBR5 associated with YWHAZ may influence prognosis in patients with liver cancer, and that UBR5 may be a candidate treatment target for liver cancer. Therefore, UBR5 associated with YWHAZ may influence prognosis in patients with liver cancer, and UBR5 could serve as a potential target for liver cancer treatment.

## Introduction

Cancer of the liver is one of the most common digestive system cancers in the world, and it is the fourth leading cause of cancer-related death in the world [1]. It is our primary focus to study hepatocellular carcinoma, a pathogenic type of liver cancer that accounts for more than 750,000 new cases and 700,000 deaths worldwide each year. In spite of significant advances in clinical diagnosis and treatment in recent years, the prognosis for liver cancer remains unsatisfactory [2]. There is an urgent need to identify important genes related to the prognosis of liver cancer patients.

Ubiquitin-proteasome system (UPS) regulates many cellular functions and is closely linked to the development of illness [3]. A number of diseases, including cancer, cause ubiquitin ligase to malfunction, which plays an essential role in the enzyme system that controls ubiquitin chain specificity and structure [4]. UBR5, an E3 ubiquitin and nuclear phosphoprotein, is found on chromosome 8q22 and is also known as human ubiquitin protein ligase E3 component N-recognin 5 [5]. UBR5 plays a role in DNA damage responses, metabolic functions, cell proliferation, and cell death. A proteolytic cleavage of the N-terminus of proteins also reveals that they contain recognition sequences [6–8]. The E3 ligase UBR5 plays an important role in cancer and development. It has recently been discovered that the UBR5 gene is frequently expressed abnormally in a variety of human cancers, such as breast cancer, ovarian cancer, lymphoma, gastric cancer, and gallbladder cancer [9–13]. It also plays a significant role in the pathogenesis of some particular disorders. There is no clear understanding of the biological function of UBR5 in liver cancer.

In the present study, we aim to demonstrate that UBR5 expression is correlated with clinicopathological characteristics of liver cancer based on TCGA data and *in vitro* experiments. We continue to investigate the biological mechanisms regulating UBR5 in liver cancer. To clarify the molecular functions of UBR5 in liver cancer, we used analyses from the Kyoto Encyclopedia of Genes and Genomes (KEGG) and Gene Ontology (GO). In order to better understand the primary pathway of differentially expressed gene enrichment in liver cancer, a protein-protein interaction (PPI) network between differentially expressed genes was created and annotated using software from the STRING website using online tools. By using Kaplan-Meier survival analysis, we determine the clinical relevance of UBR5 in liver cancer, and we suggest that UBR5 may be a biomarker for liver cancer.

The YWHAZ protein plays an important role in tumor progression and is involved in many signal transduction pathways. There is growing evidence that YWHAZ plays an oncogenic role in multiple types of cancer [14]. YWHAZ expression and liver cancer cell proliferation were affected significantly by interference with UBR5 expression *in vitro*. Thus, providing additional evidence of UBR5 as a prognostic biomarker for liver cancer patients.

## Materials and Methods

### Data Mining and Source

A dataset for liver cancer was downloaded from The Cancer Genome Atlas (TCGA) (https://cancergenome.nih.gov/). This study provides mRNA expression data for 372 tumors and 50 normal tissues, as well as clinical information such as age, clinical stage, gender, histological grade, TNM classification, survival time, survival status, and histological type. Data acquisition and applications were conducted in accordance with TCGA Publishing Guidelines and Data Access Policy. The local ethics committee does not need to approve this application.

### Bioinformatic Analysis/Statistics

Using R software (http://www.bioconductor.org/packages/release/bioc/html), we evaluated the differential expression of UBR5-related genes in liver cancer and normal samples. Pearson’s correlation analysis was used to identify gene-to-gene correlations. In the HCCDB database (http://lifeome.net/database), 15 public datasets are available, including data on UBR5 mRNA expression in liver cancer. According to the HCCDB database, liver cancer has a high expression of UBR5 compared to adjacent normal tissues. On the basis of the UALCAN website (http://ualcan.path.uab.edu/index.html), KM survival curves were analyzed to determine which genes affected survival. SPSS 17.0 (IBM, Chicago, IL, United States) and GraphPad Prism 6 (GraphPad Software, La Jolla, CA, United States) were used. UBR5 expressions have been compared between two groups using an unpaired t-test (two-tailed), with a 95% confidence interval, and a *p* value <0.05 deemed significant. In order to perform GO analysis, we used Database for Visualization, Annotation, and Integrated Discovery (DAVID) v6.7 (Leidos Biomedical Research, Inc., Bethesda, MD, United States).

### Identification of Differentially Expressed Genes

Differentially expressed genes (DEGs) were identified. On the basis of the median score, the liver cancer samples were divided into two groups (high and low). R (version 4.0.3) and the R language package were used to test between high and low packets. For screening DEGs, FDR0.05 and |logFC|>1 were deemed statistically significant.

### PPI Network Construction and Analysis

The PPI network connecting highly enriched DEGs was constructed using the string-db.org interacting gene search engine and displayed using Cytoscape. Prior to 19 DEGs, genetic screening interaction can have a maximum of 19 nodes. Proteins with a total score of interaction with UBR5 greater than 0.8 were chosen for further investigation.

### Cell Culture

Huh7 and Hep3B cells were obtained from the American Type Culture Collection (VA, United States). Cells were all cultured in Dulbecco’s modified medium (DMEM, Gibco, New York, United States), supplemented with 10% fetal bovine serum (FBS, Gibco, New York, United States), 100 μg/ml streptomycin sulfate (Sigma-Aldrich, St. Louis, MO, United States), and 100 IU/ml penicillin G (Sigma-Aldrich) in a 5% CO_2_, 37°C, and humidified incubator.

### Transfection of Oligonucleotides

Liver cancer cells were seeded into 6-well or 24-well plates. After 24 h, the liver cancer cells were transfected with 20 nm UBR5 siRNA (GenePharma, Shanghai, China) or 20 nm NC siRNA (GenePharma, Shanghai, China). The siRNAs for UBR5 can be found in the Supplementary Table S1. In order to construct the YWHAZ plasmid, we inserted the full length of YWHAZ into the pcDNA3.1 vector. As instructed, Lipofectamine 3000 (Invitrogen, California, United States) was used to transfect related oligonucleotides and plasmids into liver cancer cells.

### Western Blot

RIPA buffer (Thermo Scientific, Waltham, MA, United States) containing a protease inhibitor cocktail (Roche, Welwyn Garden, Switzerland, United Kingdom) was used to lyse tissue specimens and cells. Thirty micrograms of protein per sample were denatured in loading buffer.Proteins were separated on a 10% or 15% sodium dodecyl sulfate-polyacrylamide gel and transferred on to a nitrocellulose membrane (Bio-Rad, Hercules, United States). A 5% non-fat milk was applied, and the membrane was incubated overnight at 4°C with rabbit anti-UBR5 and rabbit anti-YWHAZ antibodies (1:1000; Santa Cruz, sc-515494 and 15222–1-AP respectively). After washing in phosphate buffered saline tween-20 (PBST), the membranes were incubated with horseradish peroxidase-goat anti-rabbit antibody (1:3000; Sigma-Aldrich, A0545) at room temperature for 2 h, then washed again in PBST. Western blots were visualized using enhanced chemiluminescence (ECL) detection reagent with an ECL kit (Cell Signaling Technology, Danvers, MA, United States). Antibodies are shown in Supplementary Table S2.

### Immunohistochemistry Assay

We used UBR5 and YWHAZ polyclonal antibodies at a dilution of 1:500 in immunohistochemistry assays on liver cancer tissue sections (UBR5: sc-9562, 1:500, Santa Cruz; YWHAZ: sc-518031, 1:100, Santa Cruz). The secondary antibody conjugated with horseradish peroxidase was applied at 37°C for 30 min, incubated with diaminobenzidine solution, and counterstained with hematoxylin.

### Cell Proliferation Assay

Cell proliferation assay was performed using MTT following the manufacturer’s instructions. 1 × 10^3^ cells were plated in each well of a 96-well plate and incubated at 37°C for 24 h. We added 10 μl MTT (50 mg/ml) to each cell culture well. Then media was removed and 100 μl DMSO was added. The OD value was measured at an absorbance of 570 nm.

## Results

### Analysis Expression of UBR5 in a Pan-Cancer

**FIGURE 1:**
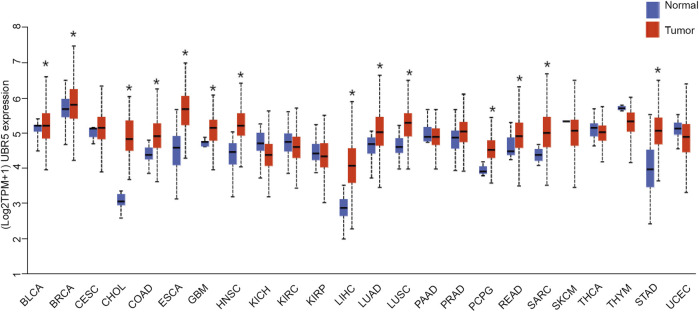
Expression of UBR5 in all TCGA patients. A boxplot displays summary statistics and individual values of UBR5 expressed in each cancer type and adjacent normal tissue. *p < 0.05.

The expression of UBR5 in tumors and normal tissues from liver cancer patients was assessed using RNA-seq data from the TCGA database. UBR5 expression was significantly increased in bladder urothelial carcinoma (BLCA), breast invasive carcinoma (BRCA), colon adenocarcinoma (COAD), esophageal carcinoma (ESCA), cholangiocarcinoma (CHOL), liver hepatocellular carcinoma (LIHC), Glioblastoma multiforme (GBM), head and neck squamous cell carcinoma (HNSC), lung squamous cell carcinoma (Figure 1). However, UBR5 expression was found in kidney renal papillary cell carcinoma (KIRP), and kidney chromophobe (KICH), kidney renal clear cell carcinoma (KIRC), uterine corpus endometrial carcinoma (UCEC), thyroid carcinoma (THCA), and thymoma (THYM) were substantially lower in cancer tissues than in non-carcinoma tissues. Based on these findings, UBR5 is implicated in the development of liver cancer, as previously found in other cancers.

### Association Between UBR5 Expression Levels With Survival and Clinicopathological Characteristics in Liver Cancer

**FIGURE 2:**
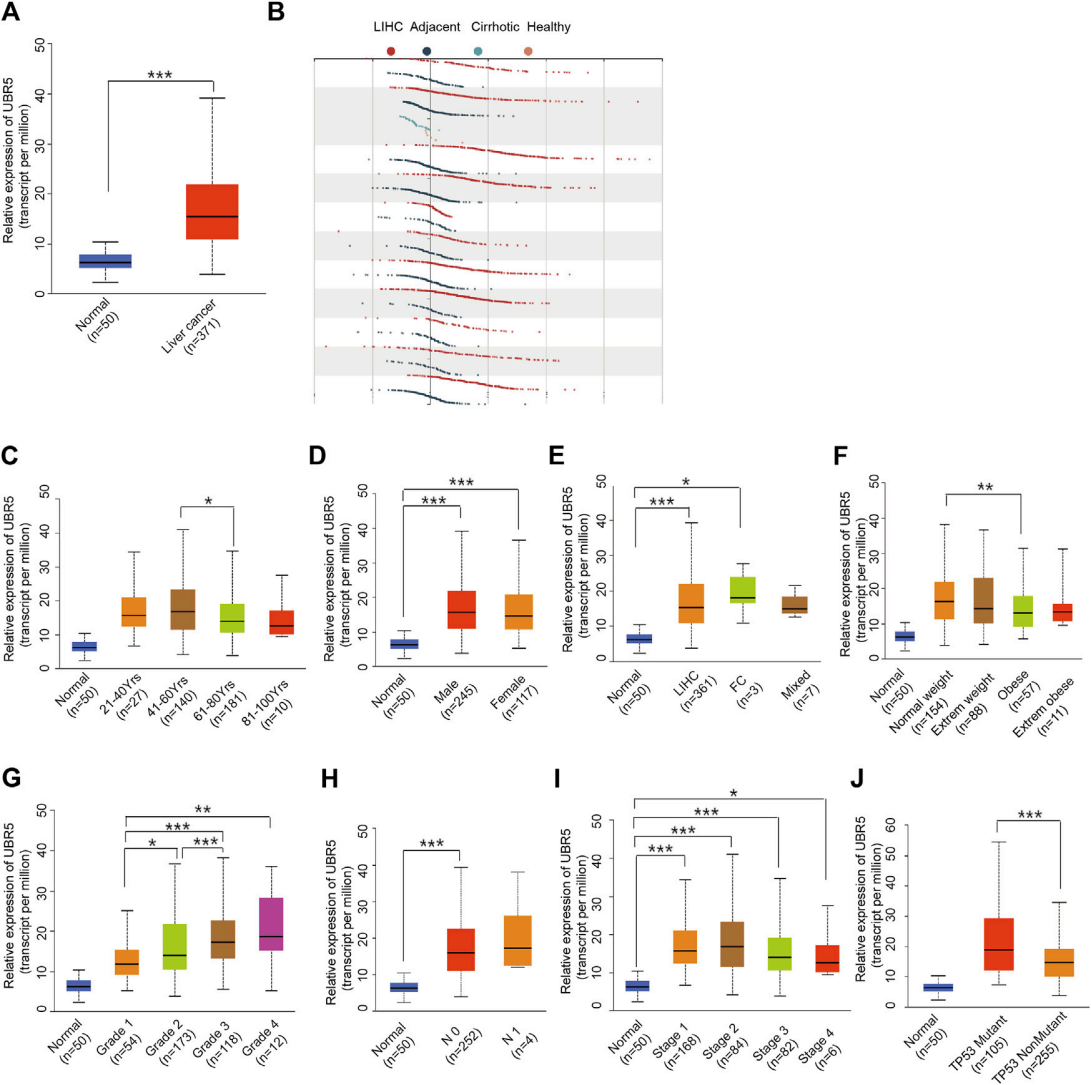
Association between UBR5 expression and clinical characteristics. **(A)** TCGA data: Expression of UBR5 in liver cancer tissues and adjacent normal tissues. Tumor tissues expressed relatively high levels of UBR5 compared to normal tissue controls. Verification in the HCCDB database **(B)**. **(C–J)** Box images for clinicopathologic characteristics such as **(C)** age; **(D)** gender; **(E)** Tumor histology; **(F)** Weight; **(G)** Histologic grade; **(H)** Nodal metastasis status; **(I)** Pathologic stage; and **(J)** TP53 status, demonstrated significant association with UBR5 expression. FC, Fibrolamellar Carcinoma; Mixed, Hepatocholangio Carcinoma. *p < 0.05, **p < 0.01, ***p < 0.001.

**FIGURE 3:**
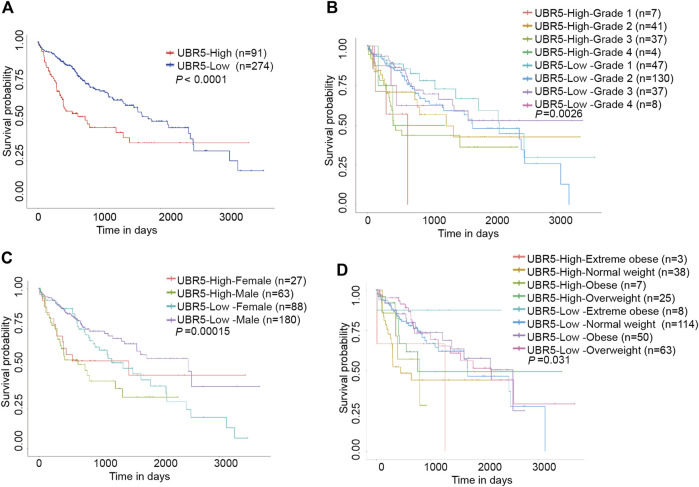
Kaplan-Meier survival curve with the Log rank test was conducted to analyze the overall survival of patients with liver cancer. **(A)** Patients with high UBR5 expression live longer than those with low UBR5 expression (P0.0001). **(B–D)** High UBR5 expression was related with poor overall survival (OS) outcomes in a TCGA cohort of liver cancer patients with clinicopathological variables such as: **(B)** histologic grade (*p* = 0.0026); **(C)** gender (*p* = 0.00015); and **(D)** weight (*p* = 0.031).

Next, we investigated whether UBR5 expression was correlated to survival and clinicopathological features in liver cancer. The TCGA data showed that UBR5 was significantly expressed in liver cancer cohorts (Figure 2A). Furthermore, an investigation of 11 liver cancer groups in the HCCDB database revealed that UBR5 mRNA expression was considerably higher in 9 of the 11 groups including HCCDB1 (GSE22058), HCCDB3 (GSE25097), HCCDB4 (GSE36376), HCCDB6 [GSE14520(GPL3721 Subset)], HCCDB13 (GSE63898), HCCDB15 (TCGA-LIHC), HCCDB16 (GSE64041), HCCDB17 (GSE76427) and HCCDB18 (ICGC-LIRI-JP) when compared to adjacent tissues (Figure 2B, Supplementary Table S3). According to these findings, UBR5 expression is increased in liver cancer. Several clinicopathological characteristics of liver cancer patients were associated with UBR5 expression. Tumor grade (G4 vs. G1, *p* = 5.6E-03; G3 vs. G2, *p* = 5.6E−04; G3 vs. G1, *p* = 1.7E−06; G2 vs. G1, *p* = 4.7E−02), age (41–60 years vs. 61–80 years, *p* = 1.8E-02), weight (normal weight vs. obese, *p* = 9.1E-03), and TP53 mutation status (mutant vs. non-mutant, *p* = 9.1E-03) were strongly correlated with UBR5 expression (Figures 2C–J). Patients were divided into several survival subgroups according to the clinical characteristics of tumor grade, gender, Body Mass Index (BMI) and UBR5 expression. Compared to the low-risk group, the high-risk group had a lower OS rate in each subgroup (Figures 3A–D, Supplementary Figure S1). A negative correlation was observed between UBR5 expression levels and liver cancer prognosis in the study.

### UBR5 Co‐Expression Networks in Liver Cancer

**FIGURE 4:**
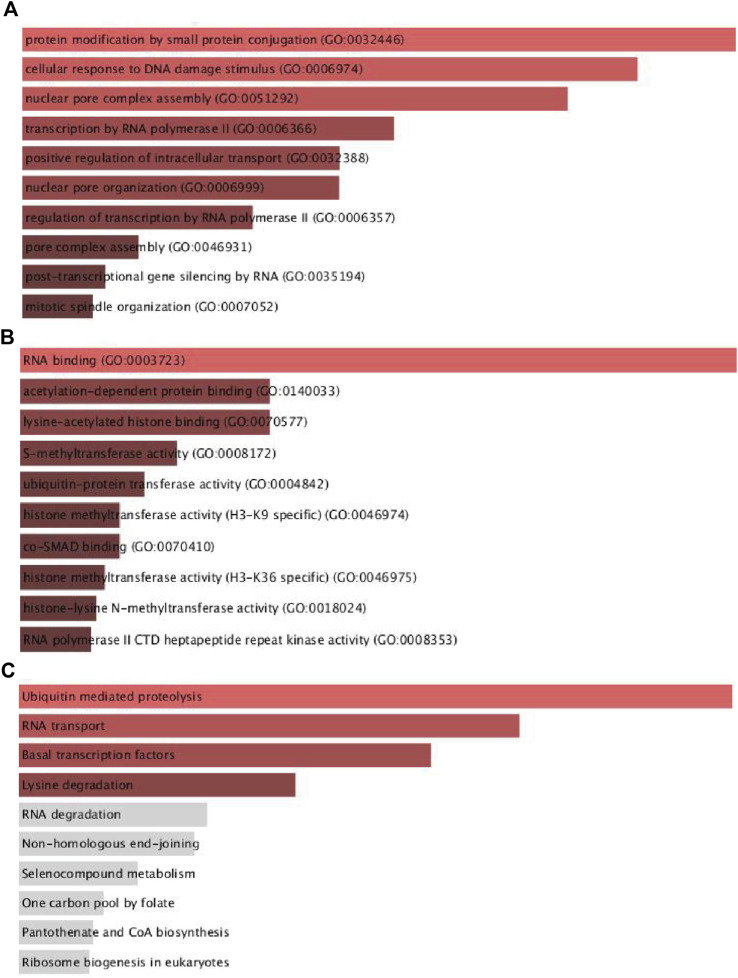
Functional enrichment analyses of UBR5 expression in TCGA-LIHC patients. **(A,B)** The top ten significant GO terms (BP/MF), and **(C)** four KEGG pathway enrichment terms of UBR5 expression in TCGA-LIHC patients. GO, Gene Ontology; KEGG, Kyoto Encyclopedia of Genes and Genomes; BP, biological process; MF, molecular function.

According to the findings presented above, UBR5 may play an important role in liver cancer. UBR5 coexpression genes were primarily involved in protein modification by small protein conjugation, cellular response to DNA damage stimulus, nuclear pore complex assembly, transcription by RNA polymeraseII, positive regulation of intracellular transport, nuclear pore organization, transcription by RNA polymerase II regulation, pore complex assembly, post-transcriptional gene silencing by RNA, and mitotic spindle organization (Figures 4A,B). According to KEGG pathway analysis (Figure 4C), these genes are primarily involved in ubiquitin-mediated proteolysis, RNA transport, basal transcription factors, and lysine degradation.

### Expression of UBR5-Related Genes in Liver Cancer

**FIGURE 5:**
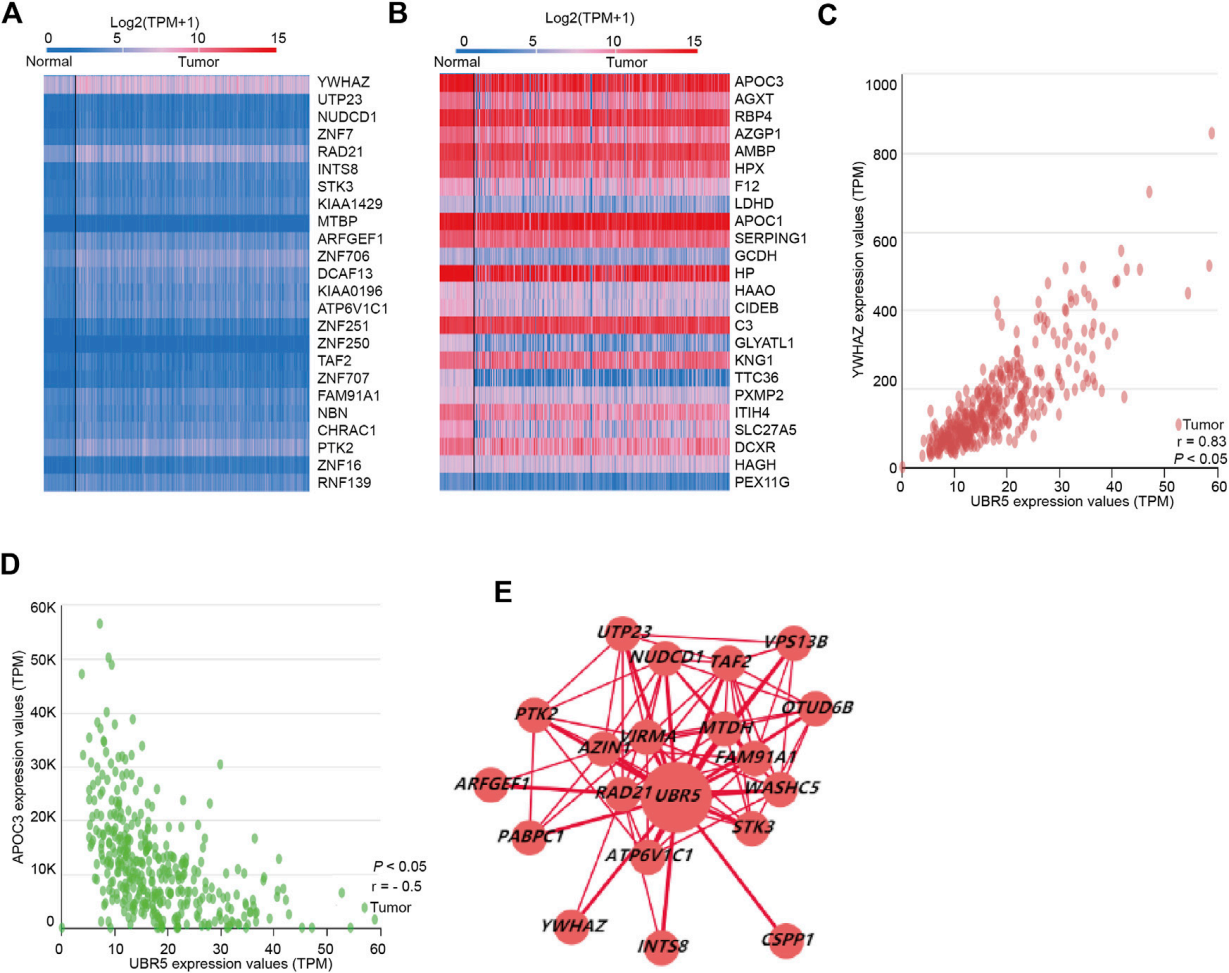
UBR5 co-expression genes in liver cancer. **(A,B)** Heat map revealed 24 important genes that were favorably and negatively associated with UBR5 respectively. **(C,D)** The pearson test was performed to identify genes in liver cancer that were differentially expressed in response to UBR5. **(E)** PPI network revealed that UBR5 had a close association with YWHAZ. PPI, protein–protein interaction.

We used linkedomics (http://www.linkedomics.org/) to examine the coexpressed genes of UBR5 in 371 patients with liver cancer. Subsequently, significant DEGs in liver cancer were found to provide further understanding of the relationship between UBR5 expression and DEGs. TCGA provides an overview of the expression of UBR5-related genes in liver cancer through the construction of a gene expression heatmap. According to the heat map (Figures 5A,B), 24 genes were positively and negatively correlated with UBR5. A total of 48 genes (24 downregulated and 24 upregulated) were identified, 24 genes were significantly upregulated in liver cancer tumors compared to adjacent normal tissue (Figure 5A). Based on Pearson correlation analysis, Figure 5B shows that YWHAZ has a positive correlation with UBR5. A correlation coefficient of 0.83 was observed between YWHAZ and UBR5 (Figure 5C). APOC3 also showed a negative correlation with UBR5, and the correlation coefficient was −0.5 (Figure 5D). Using cytoscape software, a network of PPIs was constructed between significantly enriched DEGs. A maximum number of nodes can be found before 19 DEGs through the interaction between genetic screening and genetic mapping (Figure 5E). In the TCGA database, it was found that liver tumor groups displayed significantly higher levels of YWHAZ expression than normal groups.

### YWHAZ Impairs UBR5-Suppressed Liver Cancer Cell Proliferation

**FIGURE 6:**
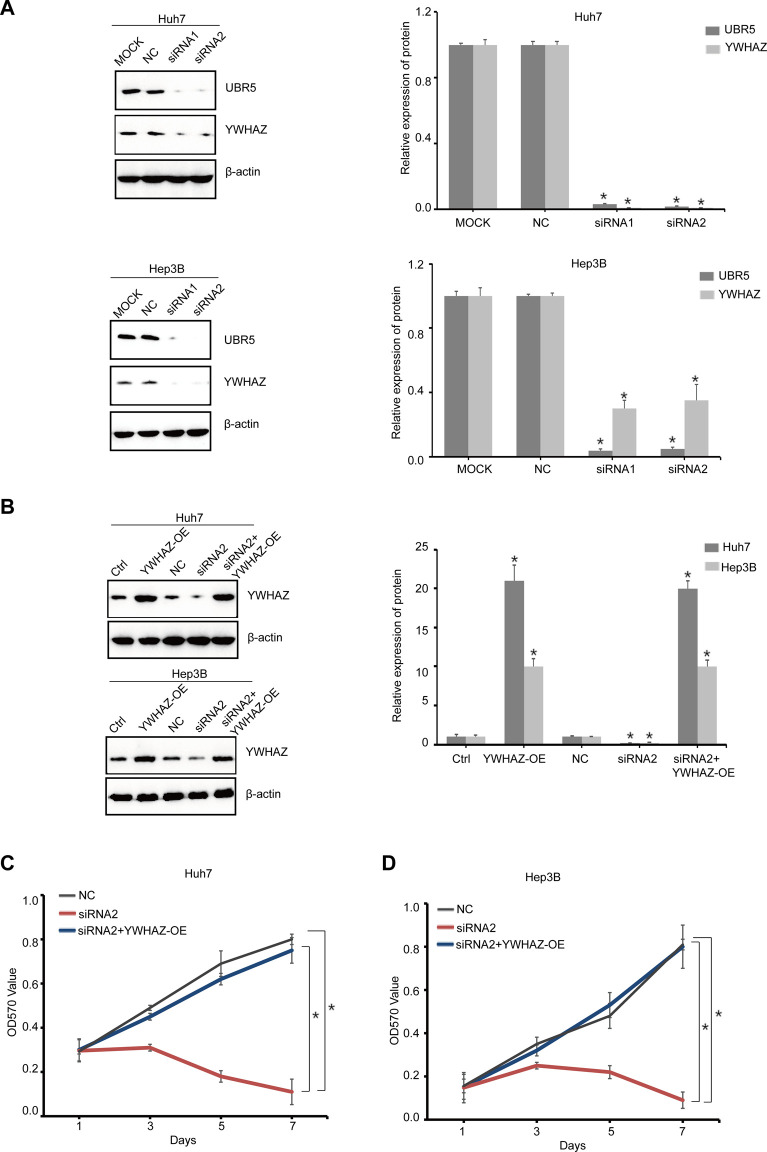
YWHAZ impairs UBR5-suppressed liver cancer cell proliferation **(A)** UBR5 and YWHAZ protein levels were detected, respectively, by western blot in Huh7 and Hep3B cells as a quality control after UBR5 knockdown (siRNA-1, 2) with siRNA *in vitro*. MOCK and NC, respectively, served as blank and negative controls. Quantification of results (right). **(B)** Western blot analysis of YWHAZ expression of YWHAZ-OE in siUBR5 (siRNA-2) transfected HCC cells or controls. Quantification of results (right) **(C,D)** cell growth at different time points (every 1 day interval) was analyzed using an MTT assay in Huh7 and Hep3B cells. YWHAZ-OE, YWHAZ Overexpression. *p < 0.05.

Transient transfection experiments were used to knock down UBR5 expression in HCC cell lines. When compared to a negative control, UBR5 siRNA treatments reduced cell proliferation in Huh7 and Hep3B cells (Figures 6A–D). Transfecting a YWHAZ expression plasmid into UBR5-suppressed liver cancer cells restored YWHAZ expression. In a functional rescue experiment using Huh7 and Hep3B cells, YWHAZ clearly restored UBR5-mediated inhibition of proliferation (Figures 6C,D). By increasing YWHAZ expression, UBR5 promotes liver cancer cell growth.

### Survival Analysis of YWHAZ in Liver Cancer Patients

**FIGURE 7:**
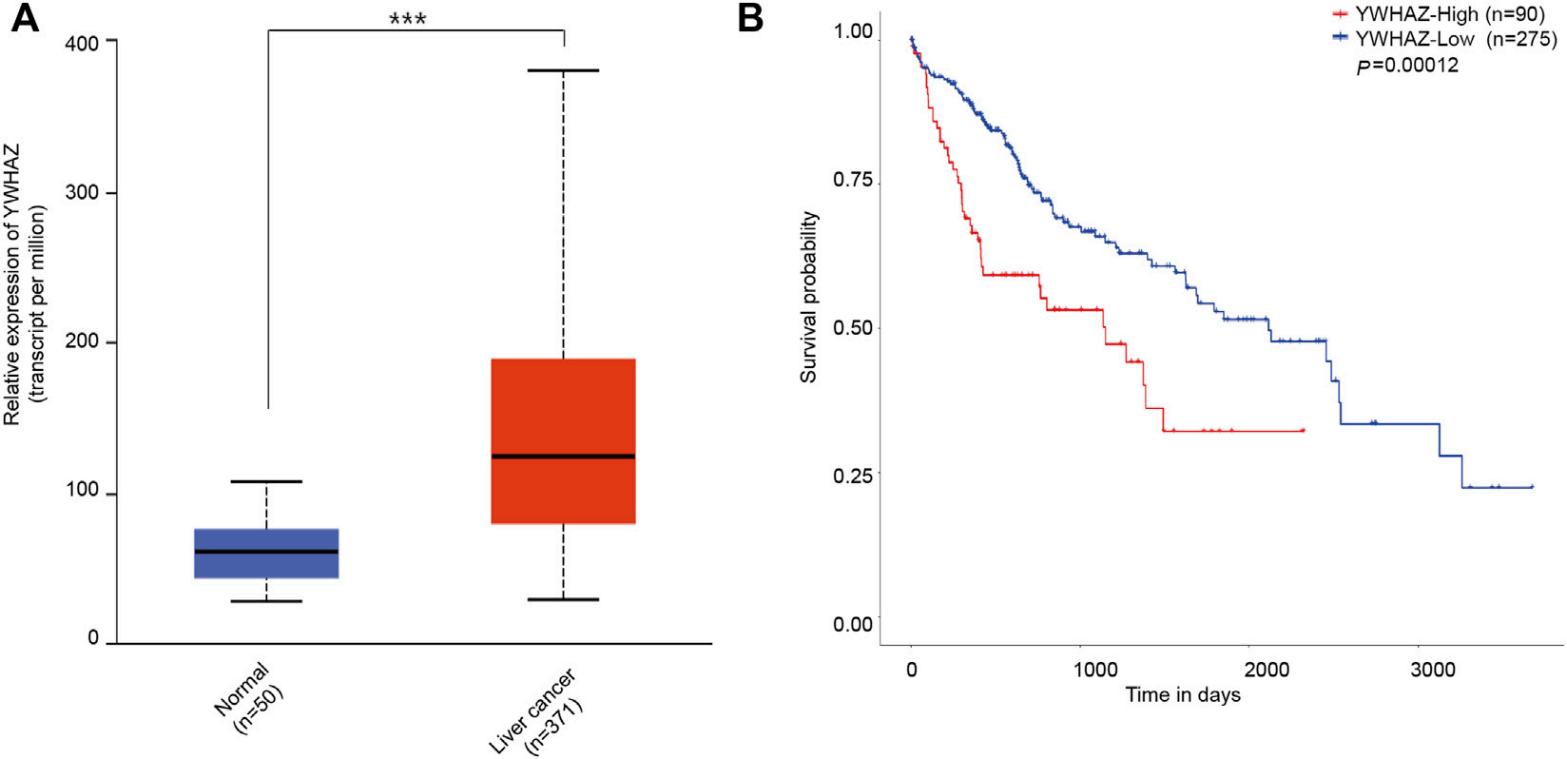
The level of YWHAZ expression in liver cancer and normal liver tissues. **(A)** YWHAZ is more abundant in liver cancer than in normal liver tissues. ***p < 0.001. **(B)** The association between YWHAZ expression and overall survival in patients with liver cancer. Kaplan-Meier survival analysis of UBR5 expression in gastric cancer patients. High YWHAZ expression was attributed to shorter survival time, while low YWHAZ expression was correlated to longer survival time (*p* = 0.00012).

In the TCGA data, YWHAZ was highly expressed in liver cancer cohorts (Figure 7A). Moreover Kaplan–Meier’s survival analysis revealed that patients with high levels of YWHAZ had shorter overall survival. Patients with high expressions of YWHAZ had significantly lower survival rates (Figure 7B). We found that our prognostic signatures were very robust at predicting OS for liver cancer patients.

### Discussion

Over the past decade, liver cancer diagnosis and treatment have improved significantly [14]. Despite widespread use of imaging in diagnosis and monitoring, liver cancer patients still have a low rate of early diagnosis. Currently, liver cancer is treated with surgical resection, chemotherapy, radiotherapy, molecular targeted therapy, immunotherapy, and TACE [15–20]. Despite continuous advancements in comprehensive treatment technology, liver cancer suffers from a poor prognosis due to its high recurrence rate and lack of effective prognostic markers. The 5-year survival rate is less than 20%. It is imperative to identify potential biomarkers in liver cancer patients in order to improve their prognoses.

The UBR box E3 ligases are the major N-recognition proteins in the N-degron pathway [21, 22]. In addition to having a wide range of substrates, these N-recognition proteins play an important physiological role [23]. UBR5 has been shown to interact with a variety of cells directly in the process of various proteins, including cell cycle, transcription, and translation mechanisms, as well as DNA damage response and repair [24]. It is crucial to understand UBR5’s role in cancer development and progression. UBR5 has many molecular functions, and it has been linked to many aspects of cancer biology. The amplification of UBR5 is a common mutation in many cancers. For example, UBR5 amplification has been associated with increased UBR5 mRNA levels in breast cancer, primarily as a result of an allelic imbalance [25, 26]. It has been shown that UBR5 is involved in therapeutic resistance in ovarian cancer [9], possibly by regulating DNA damage response. Even though some results had been reported, the role of UBR5 phosphorylation remains unclear. UBR5 has thirty-four sites of phosphorylation reported in the literature; however, little is known about UBR5 phosphorylation and how it affects protein function. UBR5 has also been identified as an unfavorable prognosis marker in pancreatic cancer [27]. According to this study, UBR5 is up-regulated in hepatocellular carcinoma and acts as an oncogene. Bioinformatics analysis is required to assess the UBR5 regulation network comprehensively. These pathways may provide important therapeutic targets for future drugs if we can better understand how N-recognition proteins are mediated in them. It is generally known that UBR5 can be mutated in patients with liver cancer. Based on the TCGA database, liver cancer patients have a mutation rate of less than 2%. UBR5 is associated with tumor grade, age, weight, and TP53 mutation status in liver cancer. Poor prognosis is associated with a high level of UBR5 expression. The findings suggest that UBR5 may be involved in liver cancer diagnosis and prognosis.

UBR5 may play a role in liver cancer diagnosis and prognosis based on these findings. Although the Supplementary Figure S3 shows significantly increased expression of UBR5 and YWHAZ in the liver tumor group compared with the normal group from clinical samples, immunohistochemistry showed that UBR5 and YWHAZ were mainly localized in the nucleus, so these findings must be confirmed in clinical liver cancer patients as a further step.

The YWHAZ gene encodes the 14-3-3 protein, which participates in several signaling pathways by binding to ligands and plays an important role in tumor genesis and development [14]. Studies indicate that YWHAZ is frequently increased in malignancies such as hepatocellular carcinoma, colorectal cancer, lung cancer, and breast cancer [28–31]. A number of biological functions are performed by YWHAZ, including cell proliferation, apoptosis, migration, and invasion [31, 32].

UBR5, as a key oncogene, is progressively being demonstrated as a biomarker for liver cancer diagnosis and prognosis.

## Data Availability

The original contributions presented in the study are included in the article/Supplementary Material. Further inquiries can be directed to the corresponding author.

## Supplementary Material

Supplementary Figure S1 Kaplan-Meier survival curve was conducted to analyze the subgroups overall survival of patients with liver cancer. High UBR5 expression was associated with poor outcomes on overall survival (OS) in liver cancer patients of a TCGA cohort with clinicopathological indicators as: **(A)** Histologic grade (G1 + G2) (*p* = 0.095) and **(B)** Histologic grade(G3 + G4) (*p* = 0.003); **(C)** Female (*p* = 0.444) and **(D)** Male (*p* < 0.001); **(D)** BMI <25 (*p* = 0.031) and BMI >25 (*p* = 0.067).

Supplementary Figure S2 The original blots of the western blot images. **(A)** UBR5 and YWHAZ protein level were detected, respectively, by western blot in Huh7 and Hep3B cells as a quality control after UBR5 knockdown (siRNA-1, 2) with siRNA in vitro. MOCK and NC were, respectively, served as blank and negative control. **(B)** Western blot analyses YWHAZ expression of YWHAZ-OE in siUBR5 (siRNA-2) trasfected HCC cells or controls.

Supplementary Figure S3 Higher expression levels of UBR5 and YWHAZ with HCC patients. **(A)** Analysis of UBR5 and YWHAZ expression in human primary HCC and adjacent non-cancerous tissues by western blot. T, liver cancer tissue; N, matched adjacent non-cancerous liver tissue. β-actin was used as an internal loading control. **(B,C)** IHC analysis of UBR5 and YWHAZ protein in liver cancer samples. Cases 1, 2, and 3, respectively, represent UBR5 and YWHAZ protein weakly, moderately and strongly stained in liver cancer tissues; original magnification for right panel: ×400).
