# Supplementary figures and images for: Clinicopathological Features and Prognostic Evaluation of UBR5 in Liver Cancer Patients
Source: Pathol Oncol Res. 2022 Nov 1;28:1610396. doi: 10.3389/pore.2022.1610396 (PMC9665233; doi:10.3389/pore.2022.1610396)

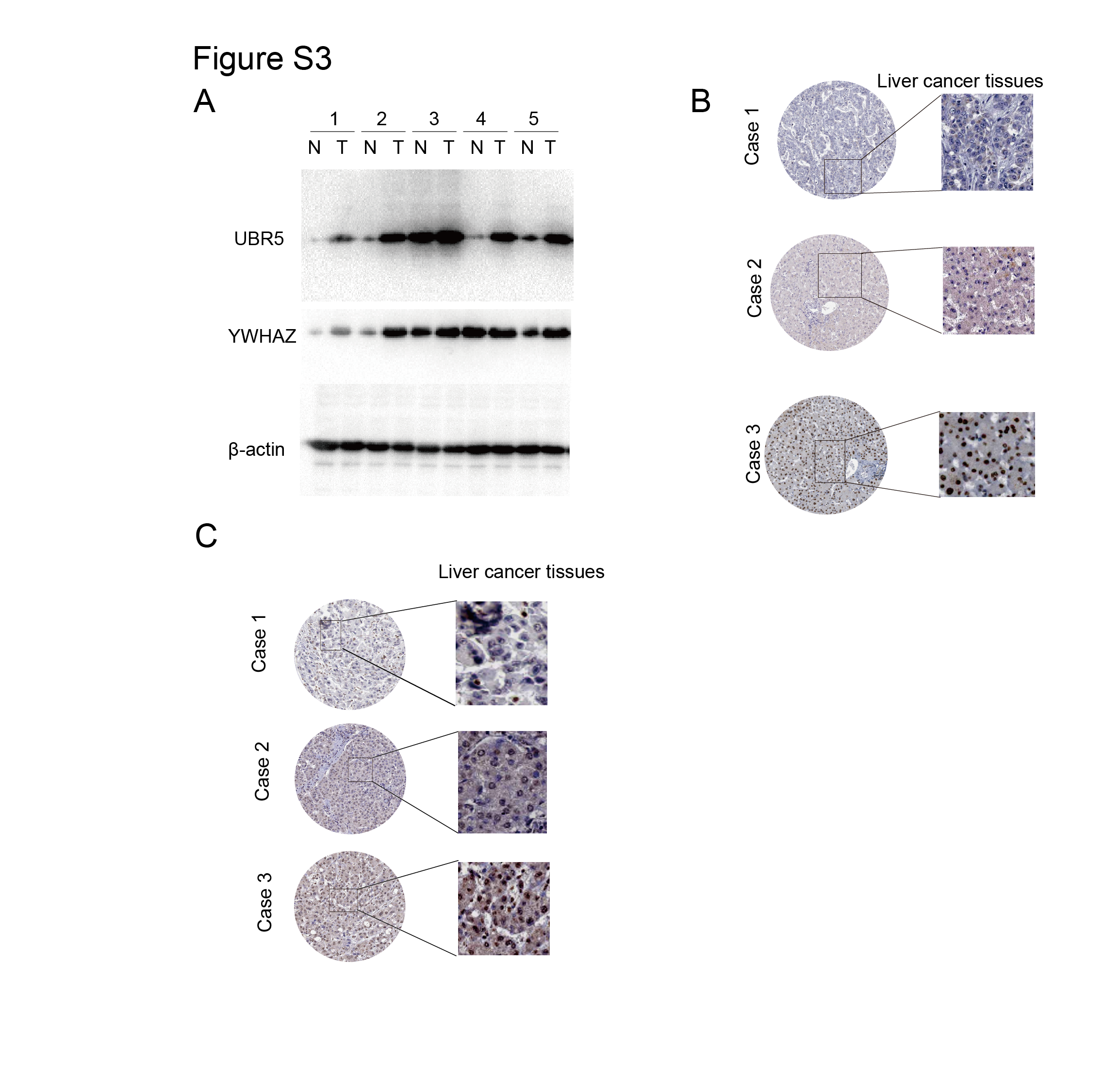

Supplement: Supplementary file 1 [file Image3.TIF]

**Table 3**

**the expression of UBR5 in the HCCDB database**


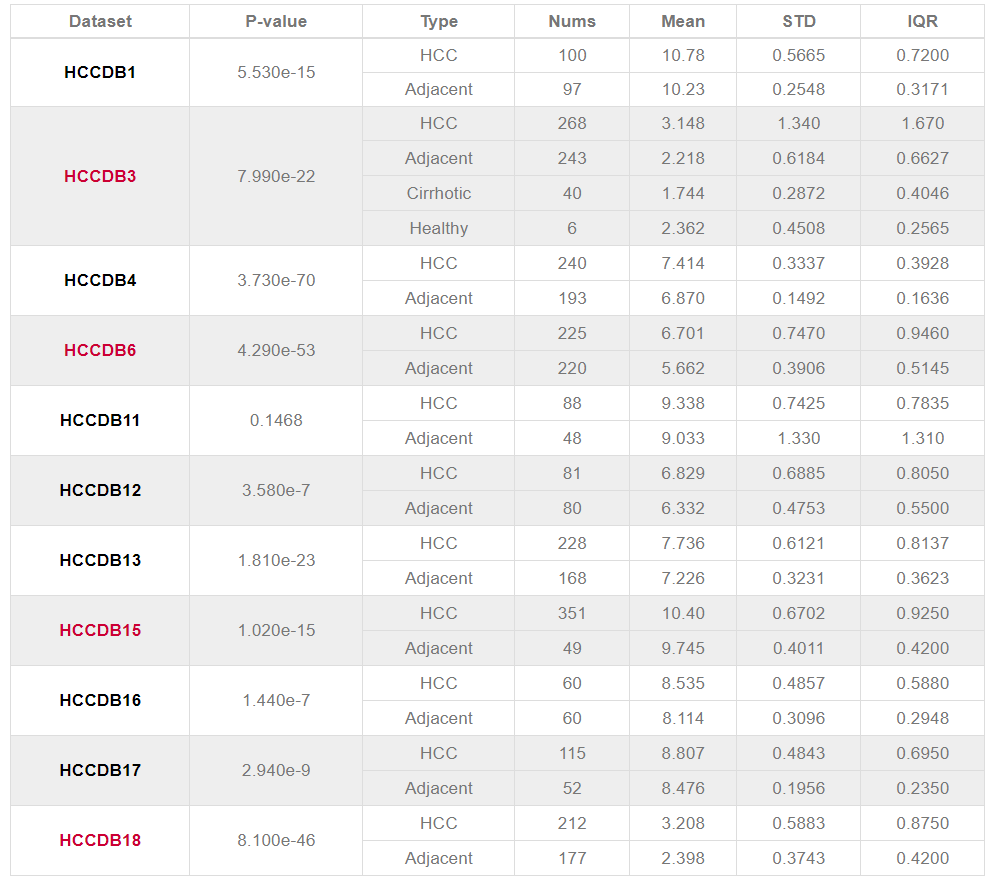

Supplement: Supplementary file 2 [file Table3.DOC]

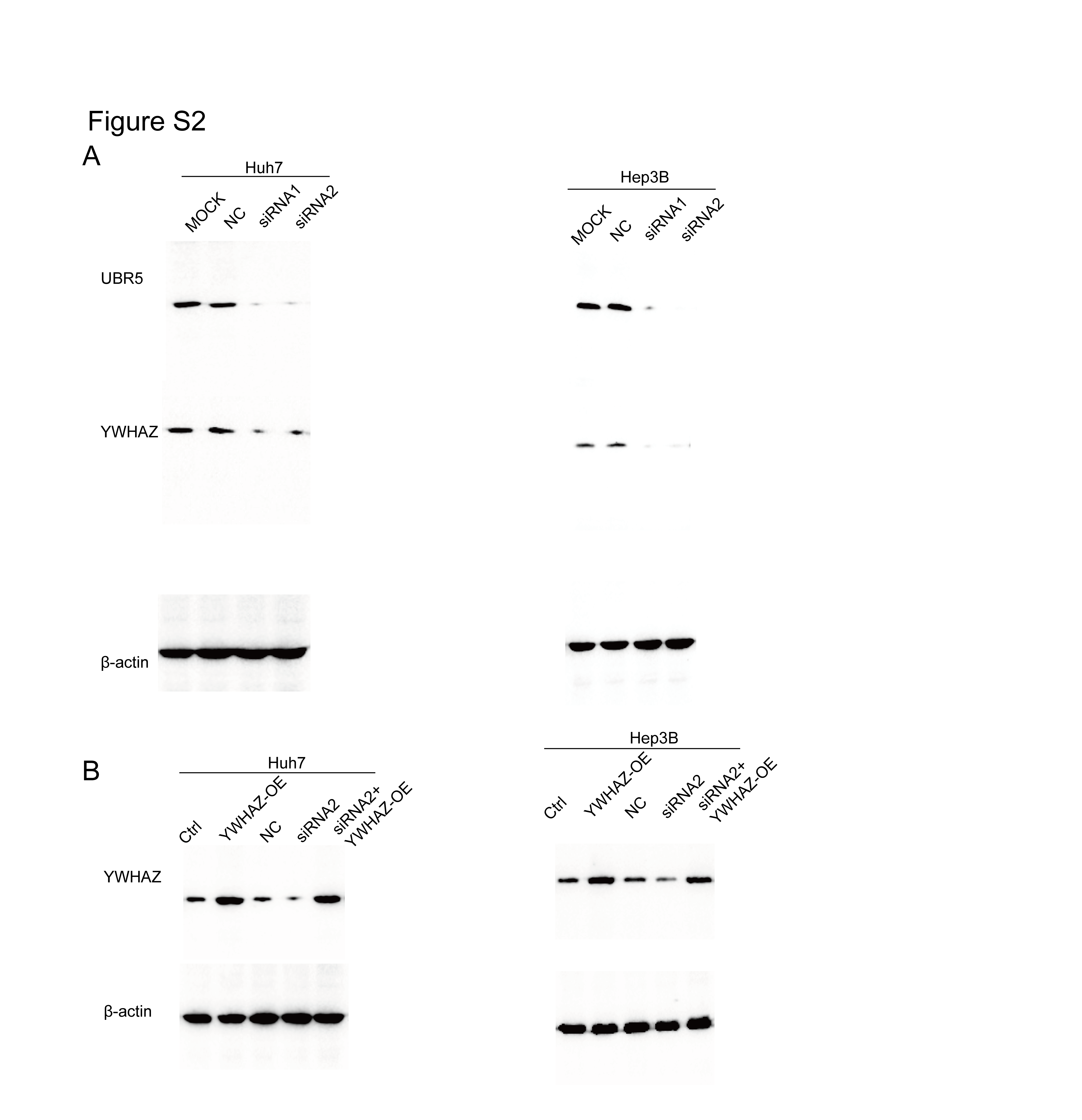

Supplement: Supplementary file 3 [file Image2.tif]

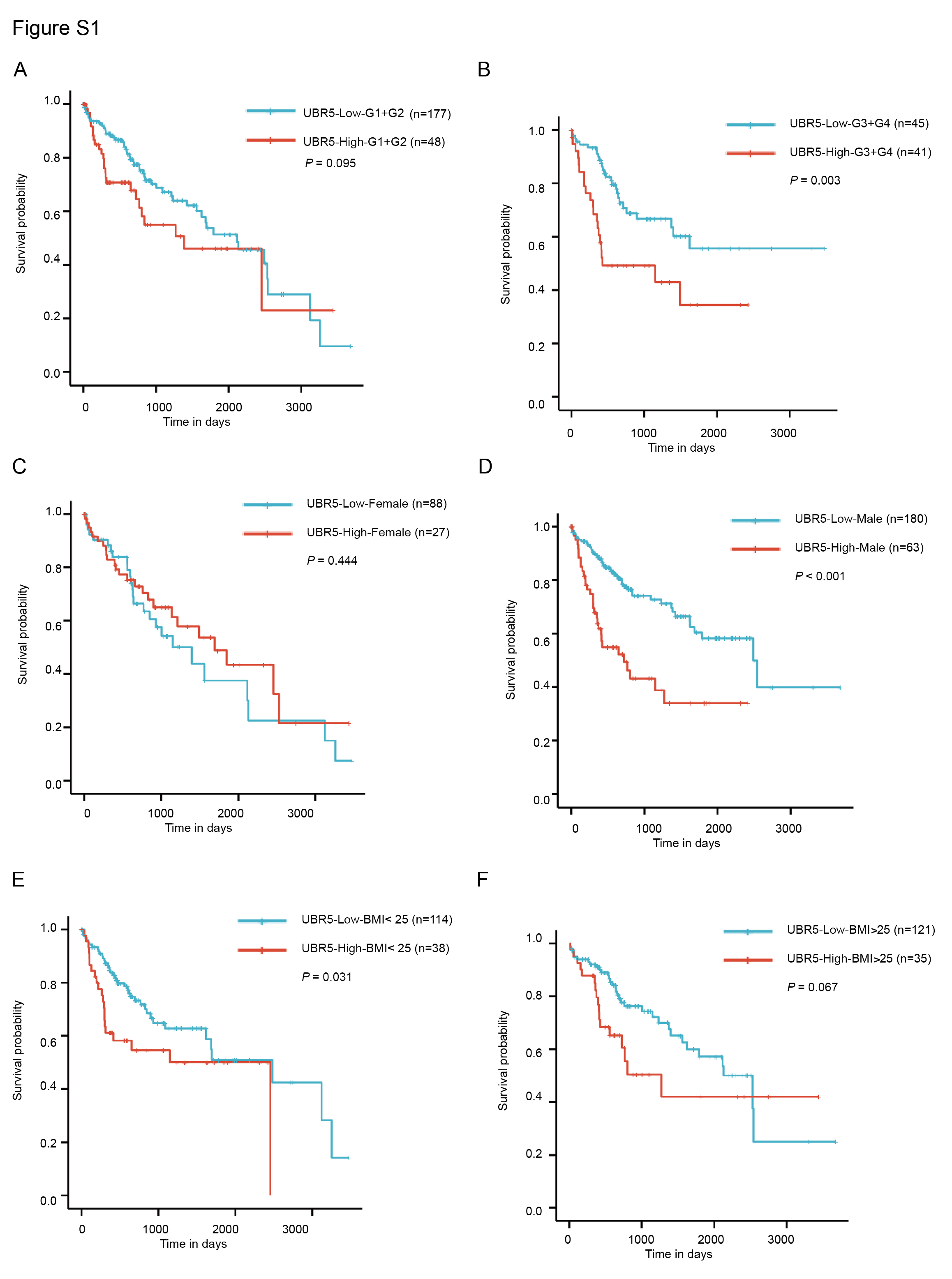

Supplement: Supplementary file 4 [file Image1.TIF]
