# Supplementary material for: Clinicopathological Features and Prognostic Evaluation of UBR5 in Liver Cancer Patients
Source: Pathol Oncol Res. 2022 Nov 1;28:1610396. doi: 10.3389/pore.2022.1610396 (PMC9665233; doi:10.3389/pore.2022.1610396)
Supplement: Supplementary file 5 [file Table1.doc]

**Supplementary material**

**Clinicopathological features and prognostic evaluation of UBR5 in liver cancer patients**

Qi Huo^1^, Junjie Hu^1^, Mei Zhao^1^, Xue Han^2^, Yulin Du^2^, Yao Li^2^*

^1^ Department of Medical Oncology, The Second Affiliated Hospital of Bengbu Medical College, Bengbu, Anhui, China,

^2^ Anhui Provincial Key Laboratory of Immunology in Chronic Diseases, Anhui Provincial Key Laboratory of Infection and Immunology, and Department of Laboratory Medicine, Bengbu Medical College, Bengbu, Anhui, China

**Corresponding Author:**

Yao Li, Electronic address: [liyao@bbmc.edu.cn](../liyao@bbmc.edu.cn), Anhui Key Laboratory of Infection and Immunity, Bengbu Medical College, No. 2600, Donghai Avenue, Longzihu District, Bengbu city 233030, Anhui Province, China

**Keywords**

UBR5, liver cancer, TCGA, YWHAZ, prognosis

**Table 1. Sequences used to silence UBR5**

| Name | Primer Sequence(5'-3') |
| --- | --- |
| SiUBR5-1 | CGAGAACUUCAGAGAACAA |
| SiUBR5-2 | CAAUGACAGGUUACGAGAA |
| NC | UUCUCCUGGAAGGUCACGU |
