# Supplementary material for: Clinicopathological Features and Prognostic Evaluation of UBR5 in Liver Cancer Patients
Source: Pathol Oncol Res. 2022 Nov 1;28:1610396. doi: 10.3389/pore.2022.1610396 (PMC9665233; doi:10.3389/pore.2022.1610396)
Supplement: Supplementary file 6 [file Table2.doc]

# Table 2. Antibodies used and dilutions

| Antibody company and catalogue number | Dilution/amount used | Application |
| --- | --- | --- |
| SantaCruz (sc-47778): β-actin | 1:2000 | WB |
| SantaCruz (sc-515494):UBR5 | 1:1000 | WB |
| Proteintech(15222–1-AP): YWHAZ | 1:500 | WB |
| Sigma A0545: Normal Rabbit IgG | 1:3000 | WB |
